# Surgical techniques for smile restoration in patients with Möbius syndrome

**DOI:** 10.4317/jced.51116

**Published:** 2013-10-01

**Authors:** Mariana Morales-Chávez, María A. Ortiz-Rincones, Fabiola Suárez-Gorrin

**Affiliations:** 1 Pediatric Dentist. Magister in Special Care Dentistry. Director of Dental Research Center. Dental School, Santa María University Caracas, Venezuela; 2 Dental Student. Santa María University. Caracas, Venezuela

## Abstract

Möbius syndrome is a congenital condition, the etiology when is not associated with misoprostol is not well defined. Signs and symptoms include difficulty swallowing, speech problems, drooling, strabismus, limitation of eye movement and more importantly, the facial blankness that these individuals have, result of the facial paralysis, due to atrophy of the cranial nerves that are involved in this condition. The ability to express emotions is affected and are considered “children without a smile.” There is currently no treatment to solvent the birth defects, the treatment options for reduce these alterations is the surgical option that has as main objective to restore muscle function through various techniques, used as required, the possibilities of applying them, is taking into consideration the outcome of the procedure to execute. Among the surgical techniques used mainly: the lengthening myoplasty of the temporal muscle,muscle transfers, cross-facial grafting, neurorrhaphy and nerve transposition, of which latter are the best performers, giving the patient a more natural, in as far as regards expression and function.

** Key words:**Möbius syndrome, surgery, smile, facial nerve, muscle transfer, transfer nerve, temporalis muscle.

## Introduction

The Möbius syndrome (MS) was first described in 1880 by von Graefe and Saemisch, and in a more complete fashion in 1888. Möbius later described it in 1892. This syndrome is quite rare around the world. The prevalence rate reported is 1/150,000 of babies born alive, although this does not take from the importance of its acknowledgement. It has to be considered that it affects the same percentage of men and women. It affects two important cranial nerves, the abducens (VI), and the facial nerve (VII), which are not completely developed, and causes facial paralysis, and limitations of ocular movement, even strabismus. These nerves control both the blinking, the lateral movement of the eyes, and the facial expressions (1,2,3,4).

Besides affecting the VI and VII cranial pair, it can also affect the glossopharyngeal (IX), and the vagus or pneumogastric (X) pairs. There are also other cranial pairs affected, such as the hypoglossal (XII), and the oculomotor nerve (III) and among the least affected nerves are the trochlear or pathetic nerve (IV), the auditory nerve (VIII), the trigeminal (V), the accessory or spinal (XI), and last, the olfactory (I). There is no evidence of the optic nerve (II) been affected by any anomaly in the development of this syndrome (1,5).

MS can be classified as classic MS, characterized by facial paralysis (60% of the cases); and as MS associated to other anomalies, whether they are other syndromes, disorders, or affectations of other nerves (40%) (2).

For the diagnosis, 5 relevant characteristics of this disease are used (1,2).

• Mask facies.

• Drooling.

• Incomplete palpebral closing.

• Convergent strabismus.

• Difficulty to swallow and speak.

Its etiology has multiple factors, and is not well defined; however, there are 4 theories considered (1,6):

1. Atrophy of the cranial nucleus, probably related to a vascular condition during the initial development of the embryo. With the interruption or alteration of the blood supply at the beginning of the development of the fetus, cranial nerve centers are damaged to different extents, which might begin with the abnormal positioning of the fetus, and the application of unusual pressure in parts of the brain that are just starting to develop. This is the most widely accepted theory.

2. The destruction or damage of the nucleus of the cranial nerves, caused by a lack of blood supply, or as a result of external effects, such as an infection (rubella), hyperthermia, generalized hypoxia and drugs. Benzodiazepines, cocaine, thalidomide and misoprostol (a synthetic analogue of prostaglandin E1 used for the treatment of peptic ulcer and for abortions), all taken during the first trimester of pregnancy, as well as gestational diabetes have been associated with the Möbius syndrome.

3. Irregularities in the peripheral nerves during development, which as a consequence lead to muscle and brain problems observed.

4. Muscles are the primary problem, and as a consequence there is a degeneration of the nucleus of peripheral nerves, and of the brain as a whole.

Facial nerve paralysis can be a disabling condition functionally, psychologically and aesthetically. Function of the facial muscles is essential for effective communication, both verbal and nonverbal, and injury to the facial nerve can severely hinder social interaction. Paralysis of the facial muscles can also result in numerous functional deficits, particularly disturbances in eye protection, eating and drinking (5,7).

Individuals suffering from MS go through several stages. The first stage takes place during the first months of life, when the feeding process is complicated and traumatic, as there are problems to swallow, choking, coughing, and vomit. Because of the danger of getting to a state of malnutrition due to the child’s rejection of food, sometimes it is necessary to practice a gastrostomy. The cause of the feeding problems comes from the damaging of nerves IX, X, and XII (1,2,5,7).

The next stage refers to facial expression (nerve VII), during which facial muscles are affected. In this phase, the child does not show any gestures or expressions when smiling or crying. Drooling starts too, and it is difficult to speak, mainly because of the damage caused to pair VII, which limits the mobility of the lips, excessive nasality (nerves IX and X), and lack of proper tongue mobility (nerve XII). There can also be a combination of the aforementioned factors (1,2,5,7,8).

As a consequence of the facial paralysis, the ability to express emotions is affected, and thus the individuals suffering from this syndrome are called “smile-less children.” Also, they are not capable of interpreting or recognizing other people’s emotions. Individuals suffering from MS cannot express the link between the neural system of imitation (mirror neurons,) and the neural system of emotions (limbic system) (7,8).

It is very particular clinic manifestation allows for the early diagnosis at birth, as the mother or the pediatrician can tell the newborn does not have a normal expression. When it cries, it produces noises and tears, but is void of any facial expression. There are also some distortions in suction, and thus it requires assisted feeding. During sleep, palpebral occlusion is incomplete. Patients later show problems producing sounds, tears, and sialorrhea, and there are also alterations for chewing, and incapacity to move the eyes sideways, which is why they have to turn their head (2).

Among the most outstanding clinical characteristics, we have (1,2,5,7,8):

• Difficulty to swallow, which can cause developmental problems; absence of smile, facial blankness.

• Sialorrhea.

• Difficulties to speak, and pronunciation problems.

• Strabismus and limitations of eye movement, cornea ulceration, and other factors associated to poor functioning of the eyelids.

• Early dental problems, such as rampant cavities or gingivitis, caused by deficient self-cleaning, inadequate diet, and the permanent half-open state of the mouth.

• Microstomia.

• Cleft palate.

• Open bite.

• Tongue atrophy.

• Mandibular hyplopasia.

These clinical characteristics are usually accompanied by (1,2,8,9):

• Anomalies in the extremities (sindactilia, polydactyly, agenesis o symbrachydactyly, clinodactyly, ectrodactyly, or rudimentary fingers, and bowlegs).

• Anomalies in the wall of the thorax, when associated to Poland Syndrome.

• Difficulty to breath.

• Mental retardation (10 - 15%).

• Associated autism (29%)

• Hearing disorder (29%).

• Corneal erosion as a consequence of the difficulty to blink, and of the corneal sensibility.

• An increase in autism symptoms has been associated to it.

• Congenital mouth anomalies, such as forked uvula, micrognathia, cleft palate, mandibular hypoplasia, maxillary hypoplasia (narrow palate), heterotopic implantation of the tongue, agenesis, tonsils hypertrophy, enamel hypoplasia, among others.

• Incapacity to make lateral and protrusive movements.

• It may be associated to syndromes such as Kallmann’s, in which there is a secondary gonadotropism; or Han-hart syndrome, in which there is agenesis of distal portions of members.

## Surgical Treatment

There is no medical treatment or cure for Möbius Syndrome. It is handled with support and reduction of symptoms, and it has to be a multidisciplinary treatment, with the participation of different health professionals, such as pediatricians, speech therapists, maxillofacial surgeons, pediatrics surgeons, children’s dentists, orthodontists, psychologists, among others. Despite several strategies for bilateral smile reconstruction that have been advocated, the condition still presents a challenge to the plastic surgeon (2,10)

Surgery could correct the eye deviation, protect the cornea through tarsorrhaphy, and improve the deformities of jaw and extremities. A so-called “smile surgery” is being performed, through muscle transfers, and grafting muscles into the mouth’s corners, in order to facilitate smiles. Although “smile surgery” can offer the ability to smile. Also, it cannot be said that this surgery cures Möbius syndrome, as it does not improve other facial expressions.

Before performing the surgical procedure, an electrodiagnosis has to be made to determine the extent of the damage, and the extension and location of the injury. Treatment for MS patients is aimed mainly at the restitu-tion or reestablishing of facial expression, facilitating smiles, reconstructing the lower lip incontinence, or oral hypotonia; and correct palpebral occlusion. In other words, restoring the protective function of the eyelids, and thus prevent corneal exposure. The ideal for this type of reestablishment of facial expression would be to restore nerve conduction through a direct suture, or through the interposition of a nerve graft, but this is not possible in every case, and several techniques have been proposed to improve facial expression through muscle transportation. Facial movements generated once such transportation is performed lack spontaneity, as it deals with several nerves, but not with facial nerves (11).

In the first half of the 20th century, various techniques for static correction with autologous temporalis muscle and fascia grafts were proposed as the techniques of Gillies [1934] and McLaughlin [1949]. Cross-facial nerve grafts have been performed since the beginning of the 1970s often with the attempt to transplant free-muscle to restore active movements. However, these transplants were non-vascularized, and further evaluations revealed central fibrosis and minimal return of function. A major step was taken in the second half of the 1970s, with the introduction of microneurovascular muscle transfer in facial reanimation, which, often combined in two steps with a cross-facial nerve graft, has become the most popular option for the comprehensive treatment of long-standing facial paralysis. In the second half of the 1990s in France, a regional muscle transfer technique with the definite advantages of being one-step, technically easier and relatively fast, namely lengthening temporalis myoplasty, acquired popularity and consensus among surgeons treating facial paralysis. Two variants of the technique (V1, V2) were developed, both based on the same anatomo-functional background and aim, which is transfer of the temporalis muscle tendon on the coronoid process to the lips (12,13).

Nowadays, this technique is one of the best surgical techniques with dynamic goals. Its more complex surgical nature, compared to previous techniques, is largely compensated by the better results obtained, and the satisfaction of the patients.

## Nerve Transfer Technique

Microsurgical technique of cross-facial nerve grafting consists in the use of interposition grafts to connect branches of the healthy facial nerve to the facial nerve of the paralyzed counter-lateral side, as described by Scaramella and Anderl [1970]. This technique allows the healthy facial nerve to send synchronized, symmetrical, and voluntary nerve impulses to the paralyzed side, which contributes to the symmetrical and spontaneous flow of movement and facial expression. The disadvantages include the long time required for reinnervation, and the limited number of donors of motor axons (10). Microsurgical reanimation is a complex, multiple, long, and expensive procedure. For these reasons, it is necessary to determine when its use is appropriate. Younger patients have the best chances of success with this type of surgery (14).

There is a third microsurgical technique called nerve transposition, used when it is not possible to perform a cross-facial nerve graft. This technique uses other ipsilateral motor nerves, in other words, from the same side as donors, to reanimate the affected side, and achieve a voluntary movement, if possible. This technique can use donor nerves, such as the hypoglossal (XII), and the motor branch of the trigeminal (V); although in the past, the spinal or accessory nerve (XI) was commonly used. Nowadays, this nerve is not used anymore. To reinnerve the affected area, these donor nerves are partially or completely sectioned, and are anastomized to the distal side of the paralyzed facial nerve, either directly or through nerve grafting. The disadvantage of this technique is that it might damage the donor nerves, thus affecting their functions. In the case of the hypoglossal nerve (XII), it could cause tongue atrophy or difficulties to swallow, fallen shoulder when the spinal nerve is affected (XI), and decrease in chewing strength, in the case of an injury of the motor branch of the trigeminal (V). Also, facial movements produced by this type of transports lack coordination if compared to the healthy side (11).

Of the aforementioned nerves, the most frequently used on patients suffering from MS, with bilateral facial paralysis, is the motor branch of the trigeminal (V), since the facial nerve (VII), and the hypoglossal (XII) are absent or non-functional. This nerve transportation technique can be used in tandem with the muscle transportation technique, which would use the gracilis muscle, and the motor branch of the trigeminal (V) to innerve such muscle (11).

In children with Möbius syndrome, bilateral facial nerve palsies preclude cross-face nerve grafts for facial reanimation. If the trigeminal nerve is functional, then the nerve to masseter represents a suitable donor nerve to innervate the functional free gracilis transfer. Coombs et al commenced using the masseteric nerve to innervate the functional free gracilis transfer in patients with Möbius syndrome in 1995. The contractions were strong and able to be controlled voluntarily. Mass movements of the face or dyskinesis were not observed. Because of the excellent results with the use of the nerve to masseter as the donor nerve for reinnervation of transferred gracilis muscles in the Möbius group of patients, combined with their own previous variable results of cross-face nerve grafts for neurotisation of gracilis muscle transfers, the masseteric nerve was also used to neurotise the gracilis motor unit in patients with unilateral facial palsy. This method has some advantages. By using a local donor nerve, the facial reanimation can be undertaken as a single procedure. It can also be done without the need for an interposition nerve graft. It became apparent that the neurovascular free muscle transfers started functioning much earlier than muscle transfers which were innervated by cross-face nerve grafts, with the average time to function of the transplanted gracilis being 9.7 weeks in their series (7).

## Muscle Transfer Technique

In 1967, Rubin was the first to report the surgical treatment for the relief of bilateral facial paralysis. By using the temporalis muscle for transplantation, with temporalis fascia and added tendons, a living motor device for activating the paralyzed upper portions of the face, including the eyelids, is provided. Bilateral transfer of the anterior third of the masseter muscles to the corners of the mouth has also been attemted. Nerve transplantation from the remaining VIIth nerve branches to the temporalis muscle transplant promises near-normal control of the facial smile (6).

In order to reproduce the zygomatic major smile, dynamic reconstructions have supplanted static, non-functioning suspension techniques when patient and anatomic factors make this feasible. In particular, functioning microneurovascular muscle transfers such as the innervated gracilis free tissue transfer, have become the method of choice over local muscle flaps in contemporary reconstruction. The most significant advantage of this option is the ability to simulate a functioning smile. A major disadvantage of this technique is its complexity, need for microsurgical expertise

and resource, and the possibility of requiring multiple stages (15).

A modified facelift type incision is used and a skin flap is elevated in the subcutaneous plane to the level of the oral commissure and extended onto the upper lip over orbicularis oris to the level of the ipsilateral philtral column. An attempt is made to identify the native zygomaticus major muscle. The elevation of the PMMT is performed as described in the cadaveric dissection. The length of the muscle from attachment to insertion is measured. Once transposed, the masseter muscle should be positioned over top of the body of the zygoma laterally and medially the muscle should reach the oral commissure in a tension free manner. This position closely approximates the normal insertion and origin of the zygomaticus major muscle. Four points of muscle fixation around the oral commissure are then identified: height of cupid’s bow on the paralyzed side, oral commissure, between the previous two points, and the lower lip slightly below the commissure. Laterally, the muscle is secured to the periosteum of the body of the zygoma (malar eminence) and infraorbital rim. A heavy permanent braided suture is used to secure the muscle at all points of fixation. Enough tension within the muscle should be created during fixation to elevate the patient’s oral commissure simulating a smile while under an esthesia (15).

## Conclusion

The Möbius Syndrome is a rare syndrome, with a 1/150 000 prevalence, that allows for different microsurgical and non-microsurgical techniques to correct the facial paralysis, and the strabismus of the patients. These techniques have suffered many modifications, in order to provide a more natural smile for the patient, and for the satisfactory result of the procedure.

According to the most recent literature, for the reestablishment of facial expression it is necessary to conduct nerve impulses through microsurgical techniques that include cross-facial nerve grafting, ideal for the cases of patients with MS suffering from unilateral facial paralysis. Nerve transposition is used when it is not possible to do the grafting due to bilateral damaging of the facial nerve.

Since it is not always possible to perform microsurgical techniques on all patients, muscle transfer is a good alternative, whether it is of the gracilis muscle, the masseter, or the temporal muscle, as long as a vascularized muscle transfer is made, and that allows for the innervation with the facial nerve, so as to obtain a more natural smile. Another widely used technique is the myoplasty of enlargement of the temporal muscle, which yields very good results at the time of reestablishing smile and facial symmetry.

All these surgical treatments have the goal of reestablishing facial expression, achieving symmetry, and facilitating the smile of the patients, which are the main advantages of the different surgical treatments; not to mention the benefits for mouth health, such as a good chewing process, and support in the development of the maxillas, if the surgery is carried out during the period of growth of the patient. It also facilitates the brushing process, and eases the job of the health specialist at the time of making certain treatments at the dental office. This is due to a better lip closing, and to a decrease in the chances of developing periodontal disease or rampant cavities. It also helps to avoid mouth breathing, pronunciation difficulties, and swallowing problems.

Most authors agree that microsurgical techniques yield better results than non-microsurgical techniques. This is because, at the time of performing the muscle transfer, other nerves are involved that make it harder for the facial expression to be as natural and symmetrical as that observed when nerve conduction is reestablished through the facial nerve.
